# Suction‐Cup‐Inspired Adhesive Micromotors for Drug Delivery

**DOI:** 10.1002/advs.202103384

**Published:** 2021-11-01

**Authors:** Lijun Cai, Cheng Zhao, Hanxu Chen, Lu Fan, Yuanjin Zhao, Xiaoyun Qian, Renjie Chai

**Affiliations:** ^1^ Department of Otolaryngology Head and Neck Surgery Affiliated Drum Tower Hospital of Nanjing University Medical School Jiangsu Provincial Key Medical Discipline Nanjing 210008 China; ^2^ State Key Laboratory of Bioelectronics School of Biological Science and Medical Engineering Southeast University Nanjing 210096 China; ^3^ Chemistry and Biomedicine Innovation Center Nanjing University Nanjing 210023 China; ^4^ School of Life Sciences and Technology Jiangsu Province High‐Tech Key Laboratory for Bio‐Medical Research Southeast University Nanjing 210096 China; ^5^ Co‐Innovation Center of Neuroregeneration Nantong University Nantong 226001 China; ^6^ Institute for Stem Cell and Regeneration Chinese Academy of Science Datun Road Beijing 100101 China

**Keywords:** adhesion, bio‐inspired, drug delivery, hydrogel, micromotors, microparticles

## Abstract

Micromotors have opened novel avenues for drug delivery due to their capacity for self‐propelling. Attempts in this field trend towards ameliorating their functions to promote their clinical applications. In this paper, an ingenious suction‐cup‐inspired micromotor is presented with adhesive properties for drug delivery in the stomach. The micromotors are fabricated by using hydrogel replicating the structure of suction‐cup‐like microparticles, which derive from self‐assembly of colloidal crystals under rapid solvent extraction, followed by loading magnesium (Mg) in the bottom spherical surface. The Mg‐loaded micromotors can realize spontaneous movement due to the continual generation of hydrogen bubbles in gastric juice. The combination of unique suction‐cup‐like structure with excellent motion performance makes the micromotor an ideal carrier for drug delivery as they can efficiently adhere to the tissue. Moreover, benefiting from the porous structure, the hydrogel micromotors exhibit a high volume‐surface ratio, which enables efficient drug loading. It is demonstrated that the suction‐cup‐inspired micromotors can adhere efficiently to the ulcer‐region in the stomach and release drugs due to their distinctive architecture and spontaneous motion, exhibiting desirable curative effect of gastric ulcer. Thus, the suction‐cup‐inspired micromotors with adhesive properties are expected to advance the development of micromotor in clinical applications.

## Graphical abstract

Herein, inspired by the suction‐cups of Octopi, an ingenious micromotor with adhesive property for drug delivery in the stomach. These micromotors can self‐drive and efficiently adhere to the wall in the harsh stomach environment for drug delivery, which are expected to advance the development of micromotor in clinical applications.

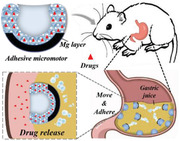

## 1 Introduction

Micromotors, which are capable of converting diverse sources of energies into propulsive force to achieve autonomous movement, have recently come to the scientific forefront as powerful simulated devices.^[^ ^1^, ^2^, ^3^ ^]^ The last decade has witnessed an unprecedented revolution in the production of micromotors because considerable endeavor has been made in the development of micromotors in various shapes based on diverse propulsion mechanisms.^[^ ^4^, ^5^, ^6^, ^7^ ^]^ Benefiting from their inherent capability of self‐propulsion, micromotors have revealed great promise in a variety of fields including environmental science, security application, biomedical engineering, and so on.^[^ ^8^, ^9^, ^10^, ^11^, ^12^ ^]^ Especially, evolved with unique features such as autonomous motion and efficient cargo loading, micromotors have proven highly advantageous as carriers for delivering drugs in vivo.^[^ ^13^, ^14^, ^15^ ^]^ In general, the micromotors loaded with drugs can react with the biofluids in vivo, resulting in propulsive force to realize autonomous movement in the complicated vivo environment, which can not only enhance the possibility of carrier‐target contacts but also prolong the retention time.^[^ ^16^, ^17^, ^18^ ^]^ Although with many successes, most of the micromotors employed as drug carriers only rely on the self‐propulsion to extend their retention time in vivo, lacking a substantial adhesion mechanism, which hinders their clinical transformation in complex biological environment. Therefore, insight regarding how to develop micromotors with adhesive properties may provide an opportunity to meet the practical requirement.

**Figure 1:**
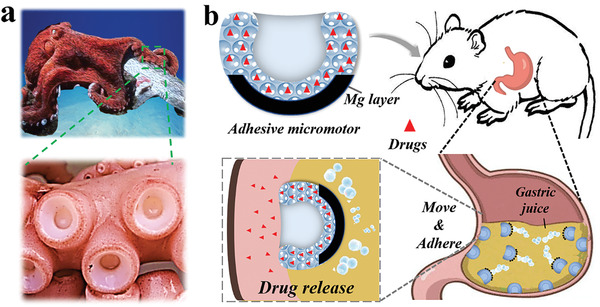
Scheme elucidation of micromotors with adhesive property. a) Pictures of octopi and their suction cups. b) Scheme of the adhesive micromotor acting as drug carries in stomach.

In this paper, inspired by the suction cup of octopi, we proposed an ingenious micromotor with adhesive property for drug delivery in the stomach, as shown in **Figure** **1**. The adhesive mechanism in nature has brought lots of inspiration to the scientists in materials science.^[^ ^19^, ^20^, ^21^, ^22^ ^]^ Octopi, which can firmly adhere to objects by taking advantage of its suction cup, has attracted great scientific attention.^[^ ^23^, ^24^ ^]^ As a result, researchers have proposed a series of bionic reversible adhesive materials with excellent performance based on the physical structure of the suction cup.^[^ ^25^, ^26^, ^27^ ^]^ Especially, films integrated with this special structure have shown broad application prospects in the fields of precision device transportation, intelligent robots, and medical treatment.^[^ ^28^, ^29^, ^30^ ^]^ However, the integration of this special structure with microparticles remains unexplored.

Herein, we fabricated the suction‐cup‐like microparticles through rapid solvent extraction of droplets containing silica nanoparticles in the microfluidics. These microparticles were then employed as templates for hydrogel to replicate the unique structure. Followed by magnesium (Mg) loaded in the bottom spherical surface, the bubble‐powered hydrogel micromotor with adhesive property was obtained. When exposed to gastric juice, the Mg‐loaded micromotors could realize spontaneous movement due to the continual generation of hydrogen bubbles derived from Mg/H+ reaction. Benefiting from the distinctive suction‐cup‐like architecture of the micromotor and the spontaneous motion, our micromotors could move and adhere efficiently to the tissue for releasing drugs, thus achieving more effective drug delivery. Moreover, owing to its porous structure, the hydrogel micromotor exhibited a high volume‐surface ratio, which enabled efficient drug loading. As proof of concept, we employed the bio‐inspired micromotors to treat gastric ulcer of mice, which indicated that the micromotors could adhere efficiently to the ulcer‐region and release drugs, exhibiting a desirable curative effect. Therefore, the bio‐inspired micromotor with adhesive property holds unpredicted potential in clinical application.

## 2 Results and Discussion

**Figure 2:**
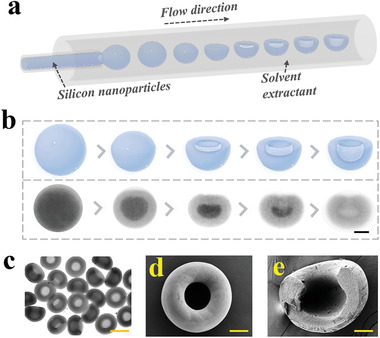
Generation and features of suction‐cup‐like microparticles. a) Illustration of the fabrication of suction‐cup‐like colloidal microparticles. b) The deformation process of the droplets during the solvent extraction. c) Images of resultant suction‐cup‐like microparticles. d,e) SEM image of the suction‐cup‐like microparticles (d), and its cross‐section (e). Scale bars are 100 µm in (b), 300 µm in (c), 100 µm in (d), and 85 µm in (e).

In a typical experiment, suction‐cup‐like microparticles were utilized as the templates for fabricating adhesive hydrogel micromotors. The suction‐cup‐like microparticles were generated from the rapid solvent extract of droplets containing silica nanoparticles in the microfluidics, where the dispersed phase was an aqueous solution of silica nanoparticles and the continual phase was rapid extractant, as depicted in **Figure** **2**a. After the dispersed phase being cut off into droplets by the continual phase, water in the droplet was extracted resulting in a rapid decrease of the diameters of the droplets (Figure 2b). Then, the speed difference between the movement of the extraction interface and the nanoparticles diffusion led to the uneven distribution of silica nanoparticles, thus resulting in the deformation of the droplets. To be specific, the top of droplets gradually folded inward and the nanoparticles self‐assembled. Finally, microparticles with suction‐cup‐like architecture were obtained after the complete extraction of water, as shown in Figure 2c. To further verify their structure, a scanning electron microscope (SEM) was employed (Figure 2d,e). Notably, the diameter of the suction‐cup‐like microparticles could be tailored by tuning the parameters of the dispersed phase and continuous phase, as shown in Figure S1, Supporting Information. It was worth mentioning that particles in microscale were more suitable for applications in vivo than that in nanoscale due to lower biotoxicity, easier production, and lower cost. More intriguingly, we could fabricate suction‐cup‐like microparticles with varied aperture ratios by tailoring the concentration of the silica colloid aqueous suspension. The aperture ratio was defined according to Figure S2a, Supporting Information. The aperture ratio of the resultant suction‐cup‐like particles reduced with the increase of the concentration of suspension (Figure S2b, Supporting Information).

**Figure 3:**
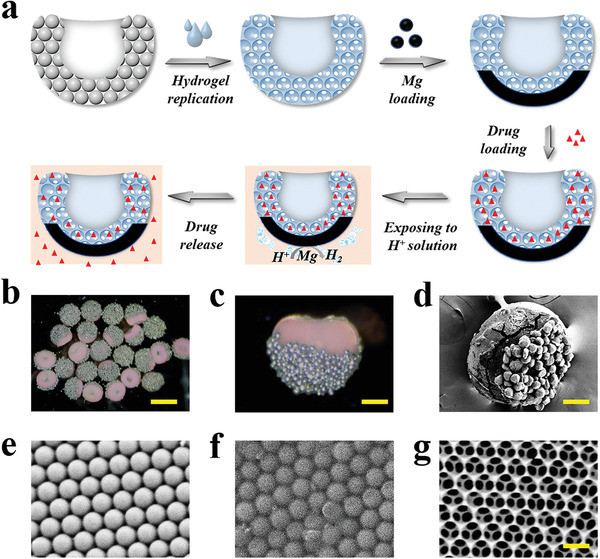
Production and figures of hydrogel micromotors. a) Elucidation of the production process of drug‐loaded hydrogel micromotors. b,c) Images of resultant adhesive hydrogel micromotors. (d) SEM image of the adhesive hydrogel micromotors. Scale bars are 300 µm in (b), 70 µm in (c), and 100 µm in (d). e–g) SEM images of the nanostructure of suction‐cup‐like microparticles (e), hydrogel‐filled suction‐cup‐like microparticles (f), and adhesive hydrogel micromotors (g). Scale bar is 280 nm.

To fabricate adhesive micromotor carriers, the resultant colloidal suction‐cup‐like microparticles were employed as templates for biocompatible hydrogel to replicate, as schemed in **Figure** **3**a. To improve the biocompatibility, methacrylated gelatin (GelMA), a kind of hydrogel with good biocompatibility,^[^ ^31^, ^32^ ^]^ was utilized to replicate the structure of the suction‐cup‐like colloidal microparticles Figure S3, Supporting Information. First, these suction‐cup‐like microparticles were pre‐dried and then soaked in a viscous melted gelatin solution. Due to its viscosity, this solution was only capable of filling the micro‐cavity of suction‐cup‐like microparticles but not the nanovoids between the nanoparticles inside. After cooling, the gelatin solidified and then gelatin outside the cavity was mechanically removed. Next, the resultant particles were saturated in the polymerizable pre‐gel solution of GelMA, which was capable of filling the nanovoids inside the particles. Subsequently, the pre‐gel was polymerized and the extra hydrogel was peered off softly, followed by immersing them in hydrofluoric acid (HF) to remove the silica nanoparticles templates. Then, the resultant hydrogel microparticles were heated to melt the gelatin inside the cavity. Finally, the adhesive hydrogel micromotors were obtained by covering the bottom of the hydrogel suction‐cup‐like microparticles with magnesium (Mg) or Zinc, biocompatible trace elements essential for many bodily functions, which can react rapidly with H^+^, resulting in rapid generation of gas bubbles. Herein, we took Mg with a diameter of about 30 µm as an example by dispersing them in a polyvidone solution and spraying them at the bottom part of the microparticles. After drying in the chemical hood for 1 h, the Mg particles were successfully loaded at the bottom of the hydrogel particles, as shown in Figure 3b,c. To further confirm this, SEM was employed (Figure 3d and Figure S3b, Supporting Information). In addition, the changes in nanostructure of the particles during the fabrication process were observed and recorded by SEM. As presented in Figure 3e, the silica nanoparticles inside the initial suction‐cup‐like colloidal particles exhibited a hexagonal close‐packed alignment. It could be seen in Figure 3f that the nanovoids between the nanoparticles were successfully filled by the pre‐gel. The final hydrogel particles exhibited a periodically ordered inverse opal structure because they were replicated from the suction‐cup‐like colloidal particles (Figure 3g).

To verify that the hydrogel micromotors can serve as drug carriers, their performance in drug delivery was tested via in vitro experiments. We employed Rhodamine B as the drug model and loaded them into the hydrogel. Notably, by simply soaking the hydrogel in the drug solution, drugs could be loaded into the carrier. Intriguingly, the nanopores inside the hydrogel particles enabled efficient‐loading of drugs by physical adsorption due to the large surface‐to‐volume ratio. The drug loading rate declined with the rising of GelMA concentration (Figure S5a, Supporting Information). To further detect the release kinetics of Rhodamine B in the hydrogel micromotor, the drug‐loaded micromotors were immersed in a simulated gastric juice oscillated at 500 rpm at 37 °C for 48 h. With the swelling and degradation of the GelMA hydrogel, the loaded drugs were released. It was found that the concentration of the GelMA had an influence on the degradation rate of the hydrogel, thus affecting the release of drugs. To be specific, GelMA hydrogel with higher concentration degraded more slowly in the simulated gastric juice (Figure S4, Supporting Information). Thus, the release kinetics of Rhodamine B from the micromotors composed of different concentrations of GelMA was recorded. As elucidated in Figure S5b, Supporting Information, it was found that there was an initial burst within the first 12 h, followed by a continual profile that finally reached the maximum. Notably, the release rate of Rhodamine B from the micromotors decreased with the raising of the GelMA concentration. Thus, for more efficient drug delivery, 10% was chosen as the final GelMA concentration for the following experiment.

**Figure 4:**
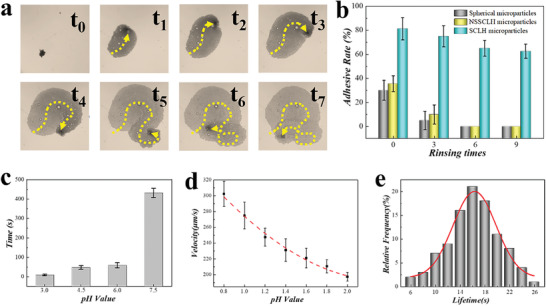
Autonomous movement and adhesive ability of micromotors. a) The time‐lapse sequence of micromotors’ movement in simulated gastric juice. b) Statistical analysis of the adhesive property of microparticles ex vivo (n = 10). c) Activation time of Mg particles in varied pH environments (n = 20). d) Statistical analysis of the velocity of micromotors in varied pH environments (n = 20). e) Statistical analysis of the lifetime of micromotors (n = 100).

To validate the adherence of our suction‐cup‐like hydrogel particles, an ex vivo attachment test was conducted. The adhesive ability of our suction‐cup‐like hydrogel microparticles was compared with that of traditional spherical microparticles by making them flow through the stomach wall of mice. Then, PBS flow rinsing was used to further confirm their adherence. It was observed that part of these microparticles adhered to the surface while others were flushed away thoroughly by the flow (Figure S6a, Supporting Information). By counting the particles that remained on the stomach wall, the adhesive rate was obtained. According to **Figure** **4**b, less than 40% of the spherical particles remained on the wall after the initial flow and they were almost flushed away after 3 times. On the contrary, 80% of our suction‐cup‐like hydrogel microparticles adhered to the wall after initial flow. And even after 9‐times rinsing, there were more than 60% of the micromotors remained, indicating that our suction‐cup‐like hydrogel microparticles exhibited good adherence to the stomach wall. In addition, the adherence of suction‐cup‐like hydrogel microparticles with different aperture ratios was also observed. Results showed that suction‐cup‐like microparticles with a 45% aperture ratio exhibited the best adherence (Figure S6b, Supporting Information). Overall, benefiting from the suction‐cup‐like morphology and nanostructure, our hydrogel microparticles possessed excellent adhesive properties.

To confirm the motional performance of the adhesive hydrogel micromotors, we observed their movement in the simulated gastric juice. Due to the presence of Mg, the micromotors were driven by the H_2_ bubbles deriving from Mg/H^+^ reaction (Figure 4a and Movie S1, Supporting Information). To be specific, the release of H_2_ bubble resulted in a counter‐force that could fuel the micromotors to move in the opposite direction and the force mainly depended on the rate of bubble formation. It was worth mentioning that the Mg/H^+^ reaction was dependent on the pH value of the surrounding solution. Faster activation could be induced by a solution with lower pH (Figure 4b). In addition, lower pH surrounding resulted in a shorter lifetime of Mg, indicating a faster reaction rate in the solution (Figure S7, Supporting Information). Because the pH value of gastric juice maintains ≈0.8–1.8 under normal circumstances, our micromotors could be propelled successfully in the gastric juice. As shown in Figure 4c, the adhesive hydrogel micromotors could move at an average speed over 200 µm s^−1^ in the simulated gastric juice, exhibiting excellent motion performance. In addition, their lifetimes were also recorded and results showed that the average lifetime of our micromotors was about 16 s (Figure 4d). Overall, our adhesive micromotor exhibited good motion performance, which paved the way for efficient drug delivery in vivo.

**Figure 5:**
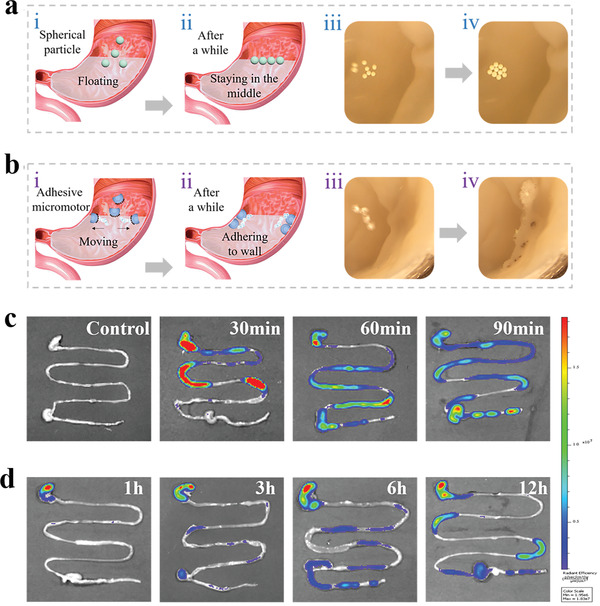
Demonstration of the gastric‐adhesive ability ex vivo and in vivo. a) Images of traditional spherical particles in the stomach ex vivo. b) Images of adhesive micromotors in the stomach ex vivo. c) The gastrointestinal tract of the mice receiving gavage of microspheres were taken out for imaging at 30, 60, and 90 mins after administration. d) The gastrointestinal tract of the mice receiving gavage of our micromotors were taken out for imaging at 1, 3, 6, and 12 h after gavage.

Considering their excellent motion performance and adhesive property, the resultant micromotors were expected to move efficiently in the complex physical environment and adhere to the tissue surface. To test this hypothesis, we observed the motion performance of the micromotors in a stomach tissue ex vivo. Besides, as comparison, we also observed the motion performance of traditional spherical microparticles. As presented in **Figure** **5**a,b and Movies S2 and S3, Supporting Information, when exposed to the simulated gastric juice, traditional spherical microparticles are suspended and kept in the middle of the stomach tissue. While our micromotors moved rapidly towards the wall of the stomach due to the generation of bubbles. After the exhaustion of the bubbles, it was found that our micromotors successfully adhered to the wall (Figure S8, Supporting Information). To further confirm their adherence, we rinsed them for several times. Results showed that even after 10 times rinsing, the micromotors still adhered to the wall, indicating excellent adhesive property due to their autonomous movement and unique structure (Movie S4, Supporting Information). Furthermore, we tested their adhesive ability in vivo. To be specific, we performed gavage of the micromotors and traditional spherical microparticles to mice, which were fasted overnight respectively. Then, the mice were sacrificed at specific time points and the gastrointestinal tract was taken out for the evaluation of adhesive properties. The retention situation could be learned by observing the fluorescence retention signal deriving from the dye inside the micromotors. It could be seen from Figure 5c, fluorescence signals of the spherical microparticles could be found both in the stomach and small intestine 30 mins after the gavage. And the fluorescence signals diffused to the distal colon in 90 mins, indicating the disability of traditional spherical microparticles for staying in the stomach with the peristalsis of digestive tract. In comparison, according to Figure 5d, the fluorescence signal could be found mainly in the stomach, revealing that our micromotors could stay in stomach for over 12 h due to their excellent adhesive property.

In view of their excellent autonomous movement, unique adhesive property, and efficient drug release ability, our adhesive micromotors were expected to serve as excellent carriers for drug delivery in vivo. To validate the practical therapeutic effect of our adhesive micromotors, treatment of mice with gastric ulcer was taken as an example. First, mice were divided randomly into five groups. Four of them were induced to gastric ulcer by acetic acid. While the other one was fed with an equivalent volume of pure water. Then, the mice with gastric ulcer received a gavage every day with PBS, non‐drug‐loaded hydrogel, free ranitidine solution, and ranitidine‐loaded hydrogel micromotors, respectively. At the same time, the healthy mice received a gavage with the same dose of PBS. Mice were sacrificed for analysis of therapeutic efficacy on day 6. As the gastric ulcer had an influence on the weight of mice, the weight of mice was recorded every day, and the weight change rate was calculated as presented in Figure S9, Supporting Information. It was found that the weight of healthy mice gained gradually. On the contrary, after the induction of gastric ulcer, the weight of mice decreased on day 2. In the following days, the weight of mice with gastric ulcer who only received gavages with PBS and non‐drug‐loaded hydrogel decreased day by day. While the weight of mice with gastric ulcer who received gavages with free ranitidine solution maintained same value as day 2 instead of decreasing and gained gradually after day 3. And the weight of mice with gastric ulcer who received gavages with ranitidine‐loaded hydrogel micromotors started an increasing trend after day 2, indicating better treatment effect of hydrogel micromotors.

**Figure 6:**
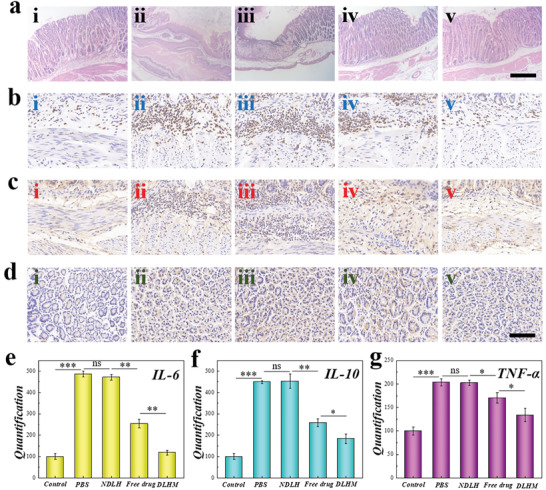
Therapeutic effect of drug‐loaded micromotor on gastric ulcer. a–d) Representative HE (a), IL‐6 (b), IL‐10 (c) and TNF‐*α* (d) images of mice of control group (i), mice with gastric ulcer that got gavage with PBS (ii), non‐drug‐loaded micromotors (iii), free drug (iv), and drug‐loaded micromotors (v). Scale bars are 300 µm in (a) and 80 µm in (b–d). e–g) Quantification of expression of IL‐6 (e), IL‐10 (f), and TNF‐*α* (e) (n = 5). (ns, none significant; *P<0.05, **P<0.01, ***P<0.001)

Furthermore, according to hematoxylin and eosin (H&E) histology images, the stomach tissue of the healthy mice was intact without inflammation, while in the stomach tissue of the mice with gastric ulcer there was an obvious loss of villi and inflammatory exudation (**Figure** **6**a). It was demonstrated inflammation was reduced more in the group of mice treated with drug‐loaded micromotors in comparison with those who received gavage with free drug and micromotors without drugs. Also, considering that interleukin‐6 (IL‐6), interleukin‐10 (IL‐10), and tumor necrosis factor‐*α* (TNF‐*α*) play a vital role in regulating inflammation, the expression of these factors in the stomach were evaluated (Figure 6b–g). Owing to the effect of IL‐6 and TNF‐*α* in promoting inflammation, they showed a high level after inducing gastric ulcer, especially in PBS group and non‐drug‐loaded micromotor group. Moreover, due to the high level of inflammation, IL‐10 was compensatively increased to suppress excessive inflammation. After 6 days, the treatment could significantly relieve the symptoms, especially the drug‐loaded micromotor group (IL‐6, P<0.01; IL‐10, P<0.05; TNF‐*α*, P<0.05). The above results indicated that the adhesive hydrogel micromotors loaded with drugs showed efficient therapeutic effects for the treatment of gastric ulcer due to their outstanding adhesion, autonomous movement, and high‐efficient drug delivery property, exhibiting their potential in clinical applications.

## 3 Conclusion

In summary, an ingenious micromotor with adhesive properties has been devised for drug delivery in the stomach. The micromotors were obtained by employing hydrogel replicating the structure of suction‐cup‐like colloidal microparticles followed by loading magnesium (Mg) at the bottom. The Mg‐loaded micromotors could realize spontaneous movement due to the continual generation of hydrogen bubbles in gastric juice, thus enhancing the retention time of carriers. Also, benefiting from their unique architecture, they possessed better adhesive ability than traditional spherical carriers. Moreover, owing to its porous structure, the hydrogel micromotors exhibited a high volume‐surface ratio, which enabled efficient drug loading. It was demonstrated that our hydrogel micromotors could adhere efficiently to the stomach wall and release drugs due to their distinctive architecture, spontaneous motion, and efficient drug release, exhibiting a desirable curative effect of gastric ulcer. Thus, the micromotors with adhesive properties were expected to advance the development of micromotor in clinical applications.

## 4 Experimental Section

### Materials

2‐hydroxy‐2‐methylacetophenone (HMPP) and Gelatin were obtained from Sigma‐Aldrich, Shanghai, China. Triacetin, HF, magnesium (Mg) particles, a tetraethyl orthosilicate (TEOS), methacrylic anhydride(MAA), polyvidone, and ranitidine were brought from Sinopharm Chemical Reagent Co., Ltd. The methacrylated gelatin (GelMA), silica nanoparticles, simulated gastric juice, and phosphate buffer saline (PBS) were self‐prepared.

### Synthesis of Silica Nanoparticles

TEOS was added to the mixture of ethanol (300 mL) and ammonium hydroxide (10 mL) with stirring (300 rpm) at 36 °C, dropwise. The nanoparticles could grow gradually due to the condensation and hydrolysis of TEOS.

### Synthesis of GelMA

8 mL MAA was added dropwise into 100 mL solution of gelatin porcine in PBS(10%, w/v) under rotation at 60 °C for 3 h. Then, PBS solution (392 mL) was added to the abovementioned mixture and kept rotating at room temperature for 15 min. After the mixture was removed into a dialysis membrane, a container filled with 5 L pure water at 50 °C was employed to hold the dialysis membrane to carry out the dialysis for 1 week. The water was changed 2 times a day. Next, a sterile filter was utilized to filter the solution. After freeze‐drying of the resultant solution, GelMA was finally obtained.

### Fabrication of Suction‐Cup‐Like Microparticles

To produce the suction‐cup‐like microparticles, aqueous solution of silica nanoparticles in different concentrations acted as the inner phase in microfluidic chips, where the outer phase was solvent extractant, the triacetin. The detailed scheme of microfluidic chips was depicted in Figure S10, Supporting Information. In detail, the flow velocity of the inner phase was 0.15 mL h^−1^ and that of the outer phase was 15 mL h^−1^. In the microfluidic chip, the formation of droplets was ascribed to the interplay of the inner phase and the outer phase. With the extraction of solvent, the droplets gradually deformed. With solvent removed, the nanoparticles gradually self‐assembled, and finally formed a suction‐cup‐like architecture and collected in a plastic box containing the extractant for ensuring all the water was extracted. To stabilize their structure, the resultant suction‐cup‐like microparticles were calcined at 800 °C in a muffle furnace for 12 h.

### Fabrication of Adhesive Hydrogel Micromotors

To fabricate the adhesive hydrogel micromotors, the resultant suction‐cup‐like microparticles were pre‐dried and steeped in a melted gelatin solution (10 wt%) at 45 °C for one hour. With the gelatin filling the cavity, the mixture was cooled for the solidification of the gelatin. After removing the extra gelatin outside the cavity by rubbing them softly with fingers, the particles were immersed in a pre‐gel solution of GelMA, with 1 vol.% HMPP. After 30 min, ultraviolet light was used to achieve the polymerization of the pre‐gel. After that, extra hydrogel was mechanically removed by rubbing them softly with fingers. Subsequently, HF (8%, v/v) was employed to remove the silica nanoparticles for 8 h, followed by heating to melt the gelation in the cavity. Then, Mg was dispersed in a polyvidone solution (4%, w/v) and spraying them at the bottom part of the microparticles. After drying in the chemical hood for 1 h, the adhesive hydrogel micromotors were obtained. The resultant micromotors were stored in a desiccator till employment.

### Establishment of Gastric Ulcer of Mice

After fasted for 24 h, the mice received a gavage of 0.5 mL double distilled water. Then, a second gavage of 0.5 mL double distilled water was conducted in 3 h. After 2 h, the mice received a gavage of 20% acetic acid solution with volume corresponding to their weight (0.025 mL/10 g).

### Drug Loading and Drug Release In Vitro

To test the performance of adhesive micromotors as drug carriers, Rhodamine B was employed as the drug model. The standard curve of Rhodamine B was detected by a microplate reader at 550 nm. The adhesive hydrogel micromotors were dried and saturated in a 1 mg mL^−1^ Rhodamine B solution for 24 h. For investigation of drug release kinetics, the micromotors loaded with Rhodamine B were immersed in 1mL of simulated gastric juice and oscillated at 500 rpm for 48 h at 37 °C. Then, 300 µL of the supernatants were sucked out to a 96‐well plate at every time interval. After that, fresh simulated gastric juice in identical volume (300 µL) was supplied to each tube. And the results were scanned by the microplate reader at 550 nm.

### Effects of Drug‐Loaded Micromotors

Mice were divided into five experimental groups randomly: i) healthy mice, ii) mice with gastric ulcer treated with PBS, iii) mice with gastric ulcer treated with free ranitidine, iv) mice with gastric ulcer treated with non‐drug‐loaded hydrogel, and v) mice with gastric ulcer treated with ranitidine‐loaded micromotors. Gavage was administered every day. Mice were sacrificed on day 6. The stomach was collected for further evaluation by HE staining and immunohistochemistry. Briefly, the isolated organs were frozen in liquid nitrogen, followed by optimal cutting temperature matrix. Afterwards, sections with a thickness of 7 µm were cut out for staining and antibodies. The histological structure of the villi and base in the stomach was then assessed.

### Characterization

Optical images of the adhesive micromotors were taken by a stereomicroscope (JSZ6S, Jiangnan novel optics) with a CCD camera (Oplenic digital camera). The SEM images of the microparticles were captured by a field emission scanning electron microscope (FESEM, Ultra Plus, Zeiss). The fluorescence pictures of particles were captured with IVIS Spectrum (PerkinElmer).

### Statistical Analysis

All the presented data were normalized on the basis of the control group. All the data were presented as mean ± SD. The sample size (n) was indicated in the figure legends of the Experimental Section. Student's t‐test was utilized to evaluate the differences between different groups, and the difference was regarded as statistically significant if *p* < 0.05. SPSS software was employed to carry out statistical analyses.

## Conflict of Interest

The authors declare no conflict of interest.

## Author Contributions

Y.Z. conceived the idea and designed the experiment. L.C. conducted experiments and data analysis, and wrote the manuscript. C.Z. conducted and analyzed the animal experiments. H.C., L.F., X.Q., and R.C. helped with the manuscript writing.

## Supporting information

Supporting Information

Supporting Movie 1

Supporting Movie 2

Supporting Movie 3

Supporting Movie 4

## Data Availability

Research data are not shared.
