# Supplementary material for: Suction‐Cup‐Inspired Adhesive Micromotors for Drug Delivery
Source: Adv Sci (Weinh). 2021 Nov 1;9(1):2103384. doi: 10.1002/advs.202103384 (PMC8728833; doi:10.1002/advs.202103384)
Supplement: Supplementary file 1 — Supporting Information [file ADVS-9-2103384-s001.pdf]

© 2021 Wiley-VCH GmbH

ADVANCED SCIENCE Open Access

Supporting Information

for *Adv. Sci.,* DOI: 10.1002/advs.202103384

Suction-cup-inspired adhesive micromotors for drug delivery

Lijun Cai, Cheng Zhao, Hanxu Chen, Lu Fan, Yuanjin Zhao, Xiaoyun Qian*, Renjie Chai**

Supporting Information

Suction-cup-inspired adhesive micromotors for drug delivery

Lijun Cai, Cheng Zhao, Hanxu Chen, Lu Fan, Yuanjin Zhao*, Xiaoyun Qian*, Renjie Chai*

Supporting figures

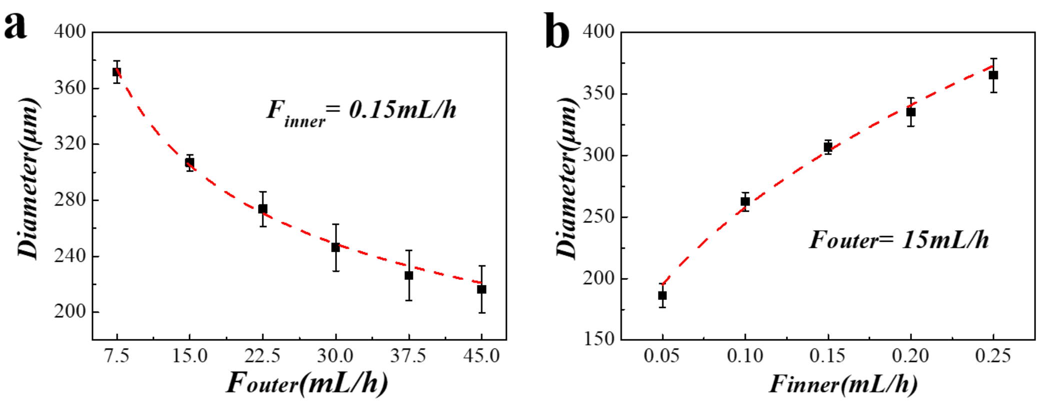

Figure S1 (a) Relationship between the particle diameter with speed of the outer phase (n=20). (b) Relationship between particle diameter with speed of the inner phase (n=20).

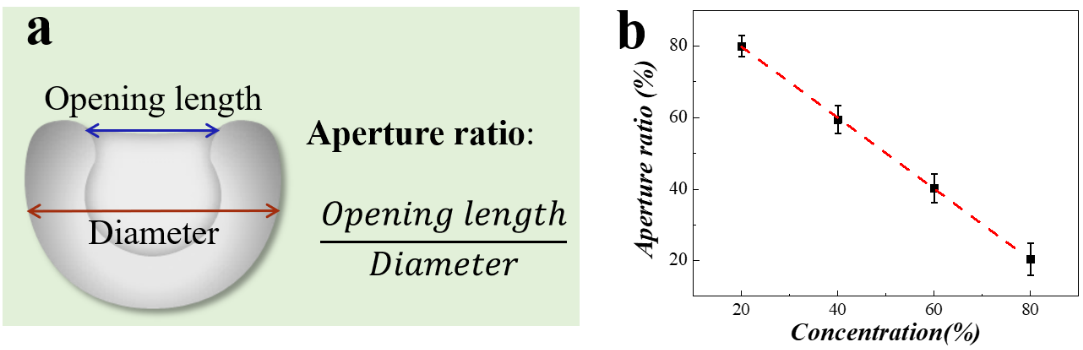

Figure S2 (a) Scheme of aperture ratio. (b) Relationship between the aperture ratio of particles with the concentrations of inner phase (n=20).

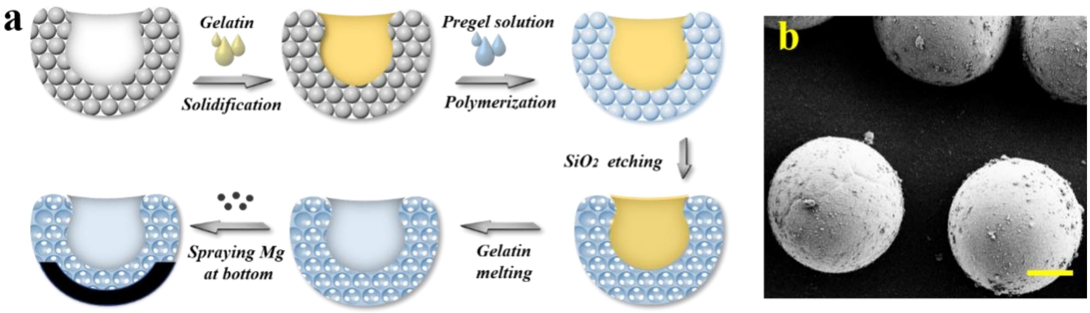

Figure S3 (a) Fabrication detail of the micromotor. (b) SEM images of the Mg particles. Scale bar is 10 μm.

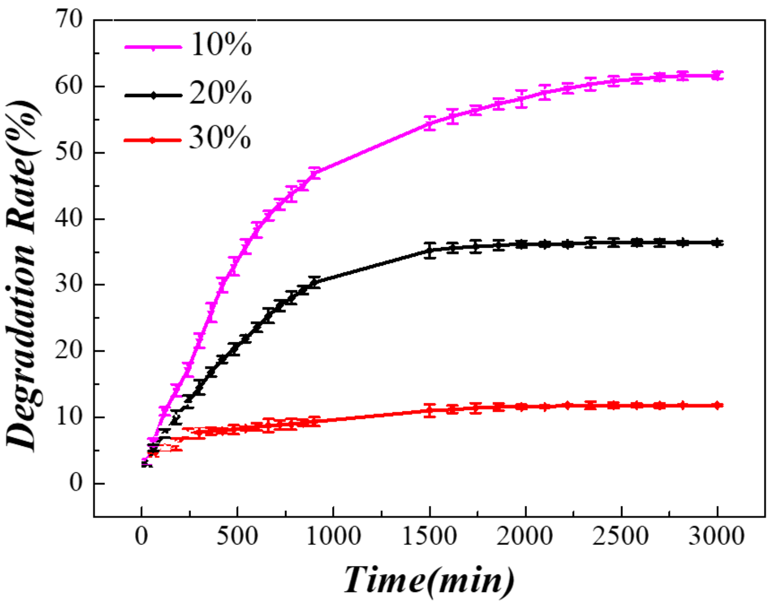

Figure S4 Degradation of GelMA in different concentration in simulated gastric juice (n=5).

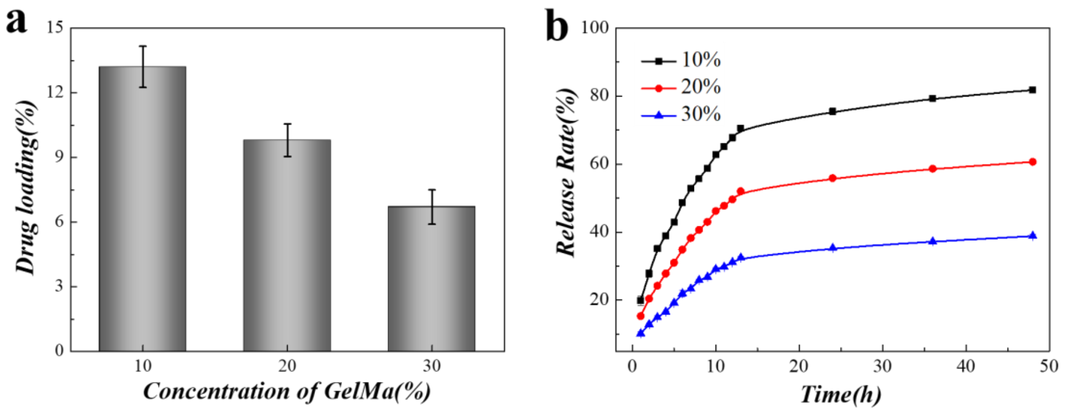

Figure S5 (a) Drug loading of GelMA in different concentration (n=5). (b) The cumulative release profiles of the hydrogel micromotors (n=5).

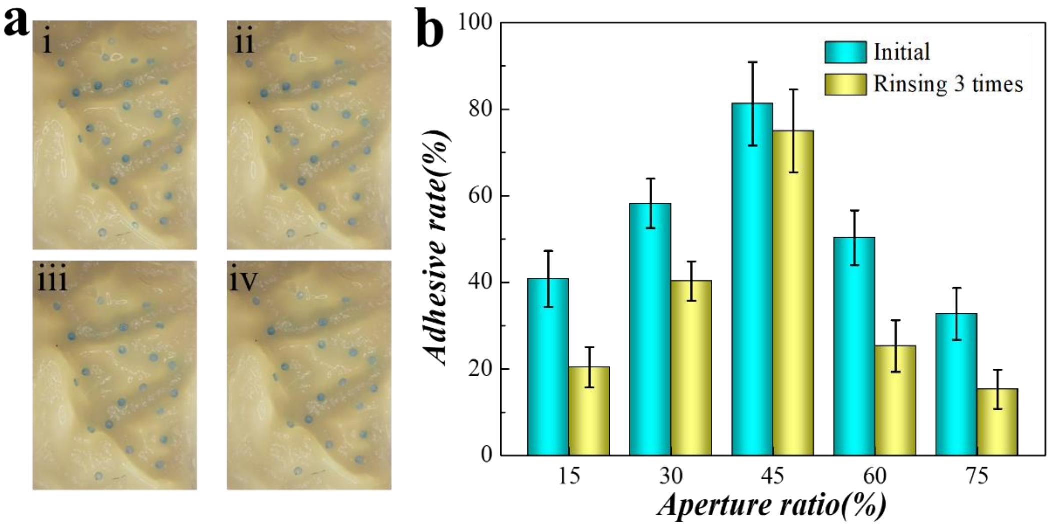

Figure S6 (a) Images of *ex vivo* adhesive test of the hydrogel after the first flow (i), rinsing 3 (ii), 6 (iii), and 9 (iv) times. (b) Statistic analysis of *ex vivo* adhesive test of micromotors in different aperture ratio (n=10).

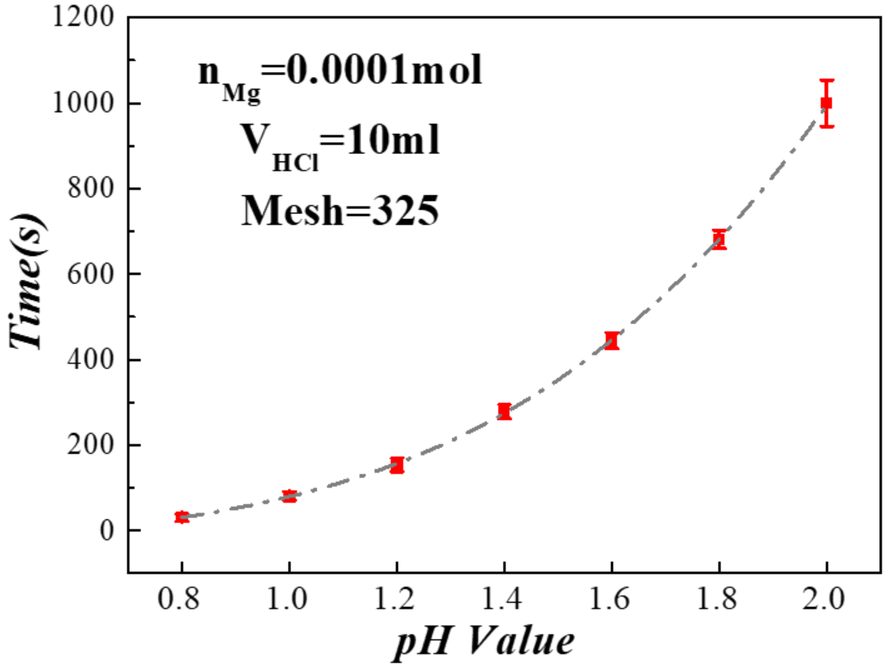

Figure S7 The lifetime of Mg in environment with different pH value (n=20).

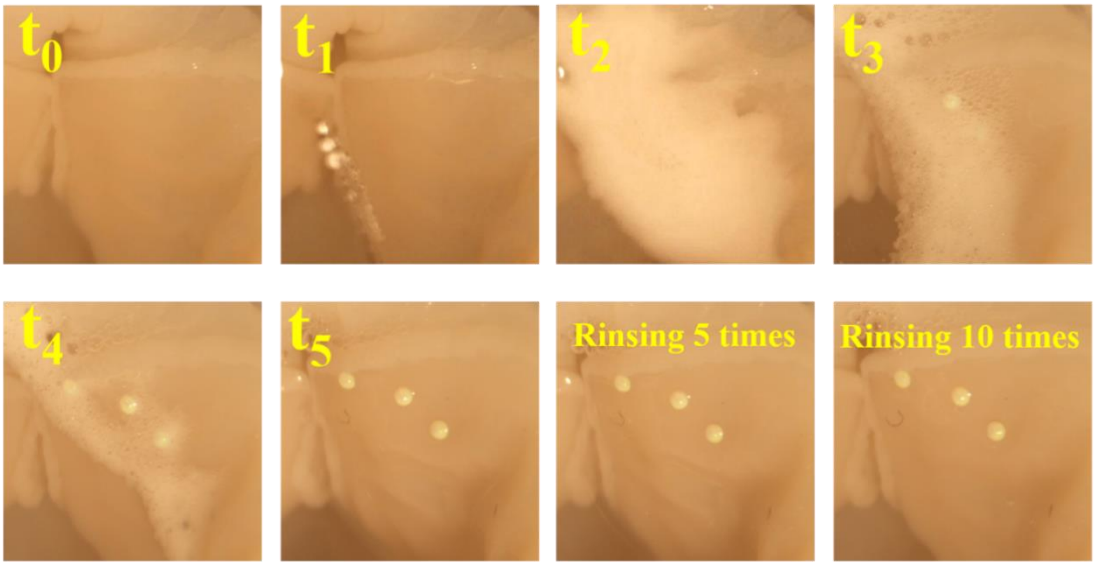

Figure S8 Images of behavior of micromotors in stomach tissue *ex vivo*.

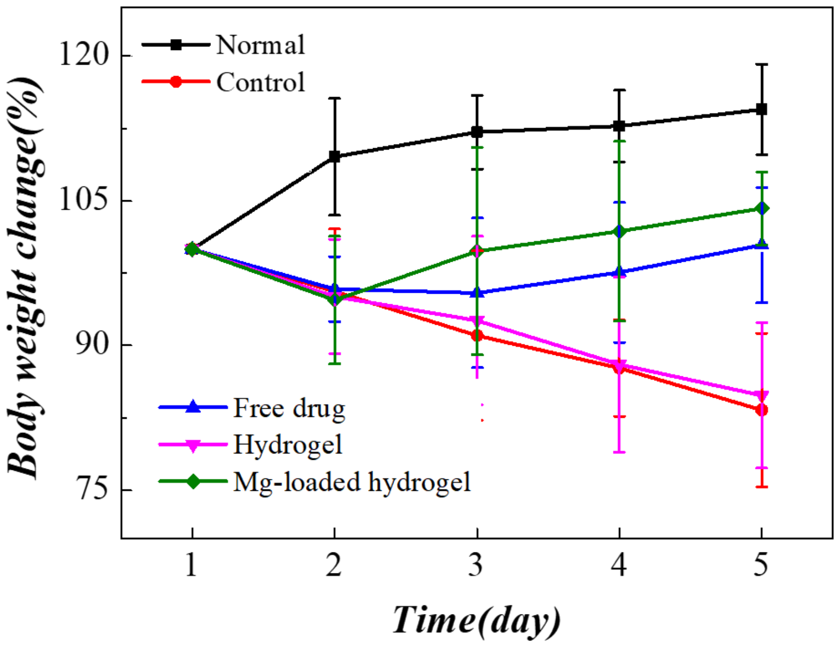

Figure S9 Statistic analysis of the weight of mice (n=5).

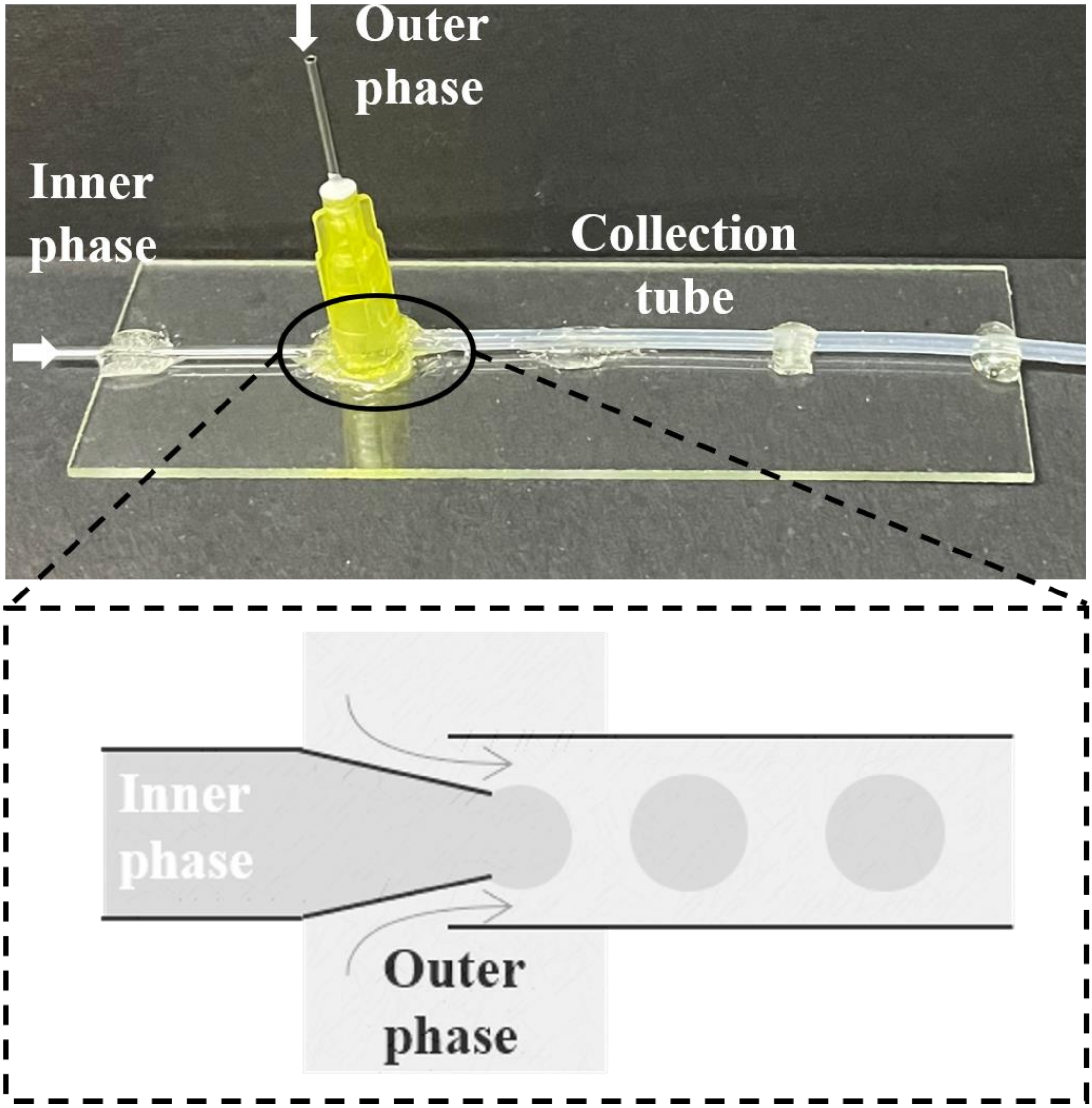

Figure S10 Scheme of microfluidic chip applied in this experiment.

Supporting movies

Movie S1 Movement of micromotors in simulated gastric juice.

Movie S2 The behavior of traditional spherical particles in stomach tissue ex vivo.

Movie S3 The behavior of micromotors in stomach tissue ex vivo.

Movie S4 Record of rinsing process.
